# 3-Cyclo­hexyl-1-(3,5-dinitro­benzo­yl)thio­urea

**DOI:** 10.1107/S1600536811013377

**Published:** 2011-04-16

**Authors:** Sohail Saeed, Naghmana Rashid, Muhammad Sher, Seik Weng Ng, Edward R. T. Tiekink

**Affiliations:** a Department of Chemistry, Research Complex, Allama Iqbal Open University, Islamabad 44000, Pakistan; b Department of Chemistry, University of Malaya, 50603 Kuala Lumpur, Malaysia

## Abstract

The structure of the title thio­urea derivative, C_14_H_16_N_4_O_5_S, features an almost planar central C_2_N_2_OS fragment (r.m.s. deviation = 0.005 Å), an arrangement stabilized by an intra­molecular N—H⋯O hydrogen bond. The terminal rings are twisted out of this plane, the dihedral angle formed with the benzene ring being 33.22 (10)°. The cyclo­hexyl ring is disordered, with two orientations (50:50) being resolved. The mean plane passing through the atoms of each disordered component forms dihedral angles of 65.7 (2) and 82.4 (3)° with the central plane. Centrosymmetric dimers mediated by an eight-membered {⋯HNC=S}_2_ synthon occur in the crystal.

## Related literature

For the biological activity of thio­urea derivatives, see: Venkatachalam *et al.* (2004 ▶); Saeed *et al.* (2011 ▶). For related thio­urea structures, see: Gunasekaran *et al.* (2010 ▶); Saeed *et al.* (2010 ▶); Dzulkifli *et al.* (2011 ▶).
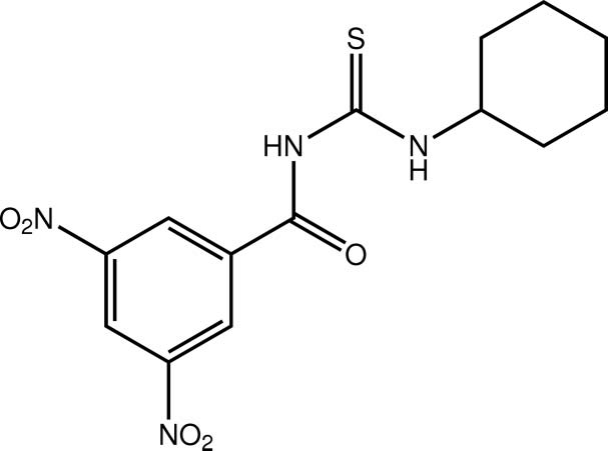


## Experimental

### 

#### Crystal data

C_14_H_16_N_4_O_5_S
*M* *_r_* = 352.37
Monoclinic, 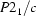

*a* = 12.3404 (7) Å
*b* = 9.0506 (5) Å
*c* = 14.6534 (6) Å
β = 90.385 (5)°
*V* = 1636.57 (15) Å^3^
*Z* = 4
Mo *K*α radiation
μ = 0.23 mm^−1^
*T* = 295 K
0.20 × 0.15 × 0.10 mm

#### Data collection

Agilent Technologies SuperNova Dual diffractometer with an Atlas detector
Absorption correction: multi-scan (*CrysAlis PRO*; Agilent, 2010 ▶) *T*_min_ = 0.955, *T*_max_ = 0.977
7954 measured reflections
3649 independent reflections
1948 reflections with *I* > 2σ(*I*)
*R*_int_ = 0.024

#### Refinement

*R*[*F* ^2^ > 2σ(*F* ^2^)] = 0.061
*wR*(*F* ^2^) = 0.211
*S* = 1.01
3649 reflections
271 parameters
25 restraints
H-atom parameters constrained
Δρ_max_ = 0.22 e Å^−3^
Δρ_min_ = −0.28 e Å^−3^

### 

Data collection: *CrysAlis PRO* (Agilent, 2010 ▶); cell refinement: *CrysAlis PRO*; data reduction: *CrysAlis PRO*; program(s) used to solve structure: *SHELXS97* (Sheldrick, 2008 ▶); program(s) used to refine structure: *SHELXL97* (Sheldrick, 2008 ▶); molecular graphics: *ORTEP-3* (Farrugia, 1997 ▶) and *DIAMOND* (Brandenburg, 2006 ▶); software used to prepare material for publication: *publCIF* (Westrip, 2010 ▶).

## Supplementary Material

Crystal structure: contains datablocks global, I. DOI: 10.1107/S1600536811013377/hb5839sup1.cif

Structure factors: contains datablocks I. DOI: 10.1107/S1600536811013377/hb5839Isup2.hkl

Additional supplementary materials:  crystallographic information; 3D view; checkCIF report

## Figures and Tables

**Table 1:** Hydrogen-bond geometry (Å, °)

*D*—H⋯*A*	*D*—H	H⋯*A*	*D*⋯*A*	*D*—H⋯*A*
N1—H1⋯O1	0.88	1.89	2.639 (4)	142
N1—H1′⋯O1	0.88	1.99	2.639 (4)	130
N2—H2⋯S1^i^	0.88	2.65	3.449 (3)	152

Symmetry code: (i) 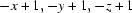.

**Fig. 1.**
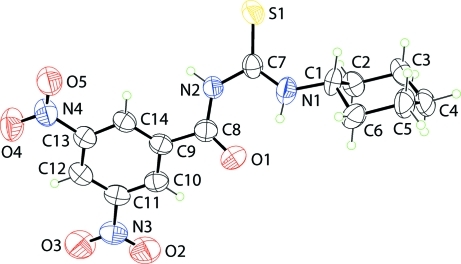
The molecular structure of (I) showing displacement ellipsoids at the 35% probability level. Only one orientation of the disordered cyclohexyl ring is shown.

**Fig. 2.**
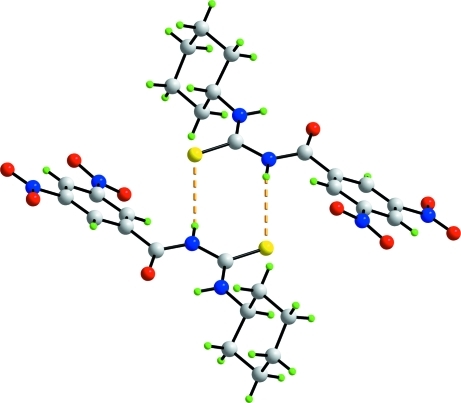
Supramolecular dimer in (I) mediated by N—H···S hydrogen bonding shown as orange dashed lines. Only one orientation of the disordered cyclohexyl ring is shown.

## supplementary crystallographic information

### Comment

Continuing structural studies (Gunasekaran *et al.* 2010; Saeed *et al.* 2010; Dzulkifli *et al.*, 2011) of thiourea derivatives are motivated by their biological potential (Venkatachalam *et al.*, 2004; Saeed *et al.*, 2011) and led to the investigation of the title compound, (I).

The molecular structure of (I), Fig. 1, is highly twisted with dihedral angles formed between the central chromophore (r.m.s. = 0.0054 Å for C7,C8,N1,N2,O1 & S1) and the benzene ring being 33.22 (10) °. Two orientations of equal weight were found for the cyclohexyl ring, each with a chair conformation, and these make angles of 65.74 (24) and 82.42 (30) °, respectively, with the central plane. The N—H atoms are anti as are the S and O atoms. As a consequence, the N1—H atom forms an intramolecular hydrogen bond with the carbonyl-O1 atom to close a pseudo six-membered ring, Table 1; there are two values cited owing to the disorder in the molecule. The nitro groups are effectively co-planar with the benzene ring to which they are bonded as seen in the values of the O2—N3—C11—C10 and O4—N4—C13—C12 torsion angles of 1.2 (5) and -7.0 (5) °, respectively.

The most prominent feature of the crystal packing is the formation of centrosymmetric eight-membered {···HNC═S}_2_ synthon leading to dimeric aggregates, Fig. 2 and Table 1.

### Experimental

A solution of 3,5-dinitrobenzoyl chloride (0.01 mol) in anhydrous acetone (75 ml) and 3% tetrabutylammonium bromide (TBAB), as a phase-transfer catalyst (PTC), in anhydrous acetone was added drop-wise to a suspension of dry potassium thiocyanate (0.01 mol) in acetone (50 ml). The reaction mixture was refluxed for 50 min. After cooling to room temperature, a solution of cyclohexylamine (0.01 mol) in anhydrous acetone (25 ml) was added drop-wise and the resulting mixture refluxed for 3 h. Hydrochloric acid (0.1 N, 300 ml) was added and the solution was filtered. The solid product was washed with water and purified by re-crystallization from ethanol; Yield: 1.50 g (88%) and *M*.pt. 409 K. IR (KBr, cm^-1^): 3215 ν(NH), 1673 (C=O), 1527 (benzene ring), 1138 ν(C═S). Anal. Calcd. for C_14_H_16_N_4_O_5_S: C, 47.72; H, 4.58; N, 15.90; S, 9.10%. Found: C, 47.51; H, 4.75; N, 15.88; S, 9.11%.

### Refinement

Carbon-bound H-atoms were placed in calculated positions [C—H 0.93 to 0.97 Å, *U*_iso_(H) 1.2*U*_eq_(C)] and were included in the refinement in the riding model approximation. The two amino H-atoms were similarly placed [N–H 0.88 Å, *U*_iso_(H) 1.2*U*_eq_(N)]. The cyclohexyl ring is disordered over two positions; the disorder could not be refined, and was assumed to be a 1:1 type of disorder. The 1,2-related C–C distances were restrained to 1.54±0.01 Å and the 1,3-related ones to 2.51±0.01 Å. The pair of *N*–C_cyclohexyl_ and *N*–*C*'_cyclohexyl_ distances were restrained to within 0.01 Å of each other.

### Crystal data

**Table:**

C_14_H_16_N_4_O_5_S	*F*(000) = 736
*M**_r_* = 352.37	*D*_x_ = 1.430 Mg m^−^^3^
Monoclinic, *P*2_1_/*c*	Mo *K*α radiation, λ = 0.71073 Å
Hall symbol: -P 2ybc	Cell parameters from 2522 reflections
*a* = 12.3404 (7) Å	θ = 2.6–29.2°
*b* = 9.0506 (5) Å	µ = 0.23 mm^−^^1^
*c* = 14.6534 (6) Å	*T* = 295 K
β = 90.385 (5)°	Prism, colorless
*V* = 1636.57 (15) Å^3^	0.20 × 0.15 × 0.10 mm
*Z* = 4	

### Data collection

**Table:**

Agilent Technologies SuperNova Dual diffractometer with an Atlas detector	3649 independent reflections
Radiation source: SuperNova (Mo) X-ray Source	1948 reflections with *I* > 2σ(*I*)
Mirror	*R*_int_ = 0.024
Detector resolution: 10.4041 pixels mm^-1^	θ_max_ = 27.5°, θ_min_ = 2.7°
ω scans	*h* = −11→16
Absorption correction: multi-scan (*CrysAlis PRO*; Agilent, 2010)	*k* = −11→9
*T*_min_ = 0.955, *T*_max_ = 0.977	*l* = −19→18
7954 measured reflections	

### Refinement

**Table:**

Refinement on *F*^2^	Primary atom site location: structure-invariant direct methods
Least-squares matrix: full	Secondary atom site location: difference Fourier map
*R*[*F*^2^ > 2σ(*F*^2^)] = 0.061	Hydrogen site location: inferred from neighbouring sites
*wR*(*F*^2^) = 0.211	H-atom parameters constrained
*S* = 1.01	*w* = 1/[σ^2^(*F*_o_^2^) + (0.0901*P*)^2^ + 0.5204*P*] where *P* = (*F*_o_^2^ + 2*F*_c_^2^)/3
3649 reflections	(Δ/σ)_max_ = 0.001
271 parameters	Δρ_max_ = 0.22 e Å^−^^3^
25 restraints	Δρ_min_ = −0.28 e Å^−^^3^

### Fractional atomic coordinates and isotropic or equivalent isotropic displacement parameters (Å^2^)

**Table:**

	*x*	*y*	*z*	*U*_iso_*/*U*_eq_	Occ. (<1)
S1	0.41365 (9)	0.64167 (12)	0.59283 (6)	0.0914 (4)	
O1	0.2058 (2)	0.5972 (4)	0.33847 (18)	0.1069 (9)	
O2	0.1940 (3)	0.2554 (4)	0.0798 (2)	0.1410 (13)	
O3	0.3315 (3)	0.2306 (4)	−0.0052 (3)	0.1351 (13)	
O4	0.6723 (3)	0.4548 (4)	0.0872 (2)	0.1237 (11)	
O5	0.6733 (2)	0.6067 (4)	0.2004 (2)	0.1140 (10)	
N1	0.2226 (2)	0.6699 (3)	0.5124 (2)	0.0864 (9)	
H1	0.1881	0.6629	0.4598	0.104*	0.50
H1'	0.1777	0.6614	0.4657	0.104*	0.50
N2	0.3606 (2)	0.5812 (3)	0.42290 (16)	0.0689 (7)	
H2	0.4294	0.5564	0.4197	0.083*	
N3	0.2883 (4)	0.2769 (4)	0.0621 (3)	0.0964 (10)	
N4	0.6288 (3)	0.5175 (4)	0.1510 (2)	0.0907 (9)	
C1	0.1543 (7)	0.7215 (9)	0.5861 (6)	0.074 (3)	0.50
H1A	0.1990	0.7735	0.6313	0.088*	0.50
C2	0.0991 (8)	0.5900 (9)	0.6317 (6)	0.084 (3)	0.50
H2A	0.1529	0.5203	0.6537	0.101*	0.50
H2B	0.0520	0.5398	0.5885	0.101*	0.50
C3	0.0323 (6)	0.6513 (8)	0.7122 (4)	0.094 (2)	0.50
H3A	−0.0055	0.5709	0.7420	0.113*	0.50
H3B	0.0807	0.6964	0.7567	0.113*	0.50
C4	−0.0488 (6)	0.7647 (9)	0.6791 (5)	0.106 (3)	0.50
H4A	−0.1009	0.7171	0.6389	0.127*	0.50
H4B	−0.0879	0.8040	0.7309	0.127*	0.50
C5	0.0049 (6)	0.8899 (8)	0.6289 (5)	0.098 (3)	0.50
H5A	0.0532	0.9427	0.6700	0.117*	0.50
H5B	−0.0496	0.9586	0.6069	0.117*	0.50
C6	0.0701 (8)	0.8278 (11)	0.5471 (5)	0.091 (3)	0.50
H6A	0.0222	0.7764	0.5051	0.109*	0.50
H6B	0.1054	0.9075	0.5146	0.109*	0.50
C1'	0.1883 (7)	0.7287 (12)	0.6020 (6)	0.121 (6)	0.50
H1B	0.2530	0.7692	0.6321	0.145*	0.50
C2'	0.1393 (7)	0.6133 (12)	0.6672 (7)	0.101 (4)	0.50
H2C	0.1891	0.5312	0.6747	0.121*	0.50
H2D	0.1277	0.6576	0.7267	0.121*	0.50
C3'	0.0326 (8)	0.5580 (10)	0.6290 (9)	0.149 (6)	0.50
H3C	0.0025	0.4846	0.6699	0.179*	0.50
H3D	0.0448	0.5112	0.5704	0.179*	0.50
C4'	−0.0484 (6)	0.6857 (12)	0.6172 (9)	0.146 (5)	0.50
H4C	−0.0635	0.7300	0.6760	0.175*	0.50
H4D	−0.1159	0.6487	0.5917	0.175*	0.50
C5'	0.0006 (8)	0.8018 (12)	0.5529 (10)	0.150 (5)	0.50
H5C	0.0107	0.7586	0.4930	0.180*	0.50
H5D	−0.0492	0.8842	0.5467	0.180*	0.50
C6'	0.1077 (7)	0.8568 (10)	0.5890 (8)	0.106 (3)	0.50
H6C	0.0968	0.9063	0.6469	0.127*	0.50
H6D	0.1376	0.9281	0.5466	0.127*	0.50
C7	0.3244 (3)	0.6318 (3)	0.5079 (2)	0.0695 (8)	
C8	0.3013 (3)	0.5664 (4)	0.3450 (2)	0.0755 (9)	
C9	0.3604 (3)	0.5071 (4)	0.2637 (2)	0.0701 (8)	
C10	0.3015 (3)	0.4227 (4)	0.2026 (2)	0.0757 (9)	
H10	0.2288	0.4025	0.2131	0.091*	
C11	0.3514 (3)	0.3689 (3)	0.1262 (2)	0.0753 (9)	
C12	0.4585 (3)	0.3965 (3)	0.1073 (2)	0.0751 (9)	
H12	0.4916	0.3582	0.0556	0.090*	
C13	0.5142 (3)	0.4833 (3)	0.1684 (2)	0.0700 (8)	
C14	0.4681 (3)	0.5400 (3)	0.2462 (2)	0.0691 (8)	
H14	0.5082	0.5988	0.2860	0.083*	

### Atomic displacement parameters (Å^2^)

**Table:**

	*U*^11^	*U*^22^	*U*^33^	*U*^12^	*U*^13^	*U*^23^
S1	0.0966 (7)	0.1080 (8)	0.0695 (5)	0.0228 (6)	0.0037 (5)	−0.0177 (5)
O1	0.0737 (17)	0.145 (3)	0.1016 (18)	0.0229 (17)	−0.0108 (14)	−0.0007 (17)
O2	0.127 (3)	0.161 (3)	0.135 (3)	−0.052 (3)	−0.019 (2)	−0.027 (2)
O3	0.144 (3)	0.126 (3)	0.136 (3)	−0.003 (2)	−0.014 (2)	−0.066 (2)
O4	0.117 (2)	0.121 (2)	0.134 (2)	−0.0209 (19)	0.045 (2)	−0.043 (2)
O5	0.108 (2)	0.131 (2)	0.1037 (19)	−0.0431 (19)	0.0195 (17)	−0.0334 (18)
N1	0.0751 (18)	0.095 (2)	0.0897 (19)	0.0212 (16)	0.0215 (15)	0.0104 (16)
N2	0.0680 (15)	0.0767 (16)	0.0620 (14)	0.0098 (13)	0.0057 (12)	0.0030 (12)
N3	0.111 (3)	0.080 (2)	0.098 (2)	−0.012 (2)	−0.019 (2)	−0.0087 (18)
N4	0.102 (2)	0.086 (2)	0.0843 (19)	−0.0157 (19)	0.0192 (18)	−0.0099 (17)
C1	0.058 (5)	0.071 (6)	0.093 (5)	0.015 (4)	0.026 (4)	0.001 (4)
C2	0.097 (8)	0.077 (5)	0.078 (6)	0.007 (6)	0.011 (6)	−0.005 (5)
C3	0.115 (6)	0.100 (5)	0.069 (4)	−0.024 (5)	0.029 (4)	−0.017 (4)
C4	0.085 (5)	0.131 (8)	0.103 (6)	−0.007 (5)	0.020 (5)	−0.071 (6)
C5	0.085 (5)	0.095 (6)	0.114 (6)	0.018 (4)	0.022 (5)	−0.037 (5)
C6	0.070 (6)	0.099 (7)	0.103 (7)	0.023 (6)	−0.007 (5)	−0.006 (5)
C1'	0.088 (8)	0.119 (11)	0.157 (11)	0.041 (7)	0.051 (7)	0.032 (8)
C2'	0.087 (7)	0.128 (8)	0.087 (7)	−0.004 (6)	0.009 (5)	0.007 (6)
C3'	0.106 (8)	0.160 (12)	0.180 (12)	−0.038 (8)	−0.043 (8)	0.069 (10)
C4'	0.071 (5)	0.162 (10)	0.203 (13)	−0.001 (7)	−0.014 (7)	0.077 (10)
C5'	0.099 (8)	0.124 (9)	0.227 (15)	−0.010 (8)	−0.057 (9)	0.049 (10)
C6'	0.090 (7)	0.092 (7)	0.136 (9)	0.007 (5)	−0.004 (6)	0.000 (6)
C7	0.076 (2)	0.0639 (18)	0.0689 (18)	0.0095 (16)	0.0127 (16)	0.0061 (14)
C8	0.077 (2)	0.076 (2)	0.073 (2)	0.0066 (18)	−0.0027 (17)	0.0090 (16)
C9	0.083 (2)	0.0665 (18)	0.0607 (16)	0.0028 (17)	−0.0076 (15)	0.0113 (15)
C10	0.078 (2)	0.0711 (19)	0.077 (2)	−0.0019 (17)	−0.0112 (17)	0.0110 (17)
C11	0.096 (3)	0.0574 (18)	0.0726 (19)	−0.0027 (18)	−0.0183 (18)	0.0045 (15)
C12	0.102 (3)	0.0593 (18)	0.0641 (18)	0.0011 (18)	−0.0014 (18)	0.0019 (15)
C13	0.083 (2)	0.0607 (17)	0.0665 (18)	−0.0054 (16)	−0.0008 (16)	0.0053 (15)
C14	0.084 (2)	0.0631 (18)	0.0598 (16)	−0.0052 (16)	−0.0035 (16)	0.0051 (14)

### Geometric parameters (Å, °)

**Table:**

S1—C7	1.659 (4)	C5—H5B	0.9700
O1—C8	1.215 (4)	C6—H6A	0.9700
O2—N3	1.210 (5)	C6—H6B	0.9700
O3—N3	1.200 (4)	C1'—C6'	1.539 (8)
O4—N4	1.221 (4)	C1'—C2'	1.542 (8)
O5—N4	1.213 (4)	C1'—H1B	0.9800
N1—C7	1.305 (4)	C2'—C3'	1.512 (8)
N1—C1	1.452 (6)	C2'—H2C	0.9700
N1—C1'	1.481 (8)	C2'—H2D	0.9700
N1—H1	0.8800	C3'—C4'	1.537 (9)
N1—H1'	0.8800	C3'—H3C	0.9700
N2—C8	1.358 (4)	C3'—H3D	0.9700
N2—C7	1.402 (4)	C4'—C5'	1.537 (8)
N2—H2	0.8800	C4'—H4C	0.9700
N3—C11	1.474 (5)	C4'—H4D	0.9700
N4—C13	1.471 (5)	C5'—C6'	1.505 (8)
C1—C6	1.524 (8)	C5'—H5C	0.9700
C1—C2	1.527 (8)	C5'—H5D	0.9700
C1—H1A	0.9800	C6'—H6C	0.9700
C2—C3	1.547 (7)	C6'—H6D	0.9700
C2—H2A	0.9700	C8—C9	1.501 (5)
C2—H2B	0.9700	C9—C10	1.380 (5)
C3—C4	1.511 (8)	C9—C14	1.388 (4)
C3—H3A	0.9700	C10—C11	1.370 (5)
C3—H3B	0.9700	C10—H10	0.9300
C4—C5	1.507 (7)	C11—C12	1.375 (5)
C4—H4A	0.9700	C12—C13	1.372 (5)
C4—H4B	0.9700	C12—H12	0.9300
C5—C6	1.552 (8)	C13—C14	1.376 (4)
C5—H5A	0.9700	C14—H14	0.9300
			
C7—N1—C1	133.3 (5)	C2'—C1'—H1B	107.3
C7—N1—C1'	114.9 (5)	C3'—C2'—C1'	109.8 (7)
C7—N1—H1	113.3	C3'—C2'—H2C	109.7
C1—N1—H1	113.3	C1'—C2'—H2C	109.7
C7—N1—H1'	122.6	C3'—C2'—H2D	109.7
C1'—N1—H1'	122.6	C1'—C2'—H2D	109.7
C8—N2—C7	127.2 (3)	H2C—C2'—H2D	108.2
C8—N2—H2	116.4	C2'—C3'—C4'	110.9 (7)
C7—N2—H2	116.4	C2'—C3'—H3C	109.5
O3—N3—O2	123.5 (4)	C4'—C3'—H3C	109.5
O3—N3—C11	119.1 (4)	C2'—C3'—H3D	109.5
O2—N3—C11	117.4 (4)	C4'—C3'—H3D	109.5
O5—N4—O4	124.5 (3)	H3C—C3'—H3D	108.1
O5—N4—C13	117.9 (3)	C3'—C4'—C5'	109.0 (7)
O4—N4—C13	117.6 (3)	C3'—C4'—H4C	109.9
N1—C1—C6	108.7 (6)	C5'—C4'—H4C	109.9
N1—C1—C2	109.8 (6)	C3'—C4'—H4D	109.9
C6—C1—C2	110.6 (7)	C5'—C4'—H4D	109.9
N1—C1—H1A	109.3	H4C—C4'—H4D	108.3
C6—C1—H1A	109.3	C6'—C5'—C4'	111.1 (7)
C2—C1—H1A	109.3	C6'—C5'—H5C	109.4
C1—C2—C3	107.2 (5)	C4'—C5'—H5C	109.4
C1—C2—H2A	110.3	C6'—C5'—H5D	109.4
C3—C2—H2A	110.3	C4'—C5'—H5D	109.4
C1—C2—H2B	110.3	H5C—C5'—H5D	108.0
C3—C2—H2B	110.3	C5'—C6'—C1'	111.1 (7)
H2A—C2—H2B	108.5	C5'—C6'—H6C	109.4
C4—C3—C2	110.7 (5)	C1'—C6'—H6C	109.4
C4—C3—H3A	109.5	C5'—C6'—H6D	109.4
C2—C3—H3A	109.5	C1'—C6'—H6D	109.4
C4—C3—H3B	109.5	H6C—C6'—H6D	108.0
C2—C3—H3B	109.5	N1—C7—N2	116.4 (3)
H3A—C3—H3B	108.1	N1—C7—S1	125.6 (3)
C5—C4—C3	112.0 (6)	N2—C7—S1	118.0 (2)
C5—C4—H4A	109.2	O1—C8—N2	124.1 (3)
C3—C4—H4A	109.2	O1—C8—C9	119.7 (3)
C5—C4—H4B	109.2	N2—C8—C9	116.2 (3)
C3—C4—H4B	109.2	C10—C9—C14	120.0 (3)
H4A—C4—H4B	107.9	C10—C9—C8	117.2 (3)
C4—C5—C6	109.7 (6)	C14—C9—C8	122.8 (3)
C4—C5—H5A	109.7	C11—C10—C9	119.2 (3)
C6—C5—H5A	109.7	C11—C10—H10	120.4
C4—C5—H5B	109.7	C9—C10—H10	120.4
C6—C5—H5B	109.7	C10—C11—C12	122.6 (3)
H5A—C5—H5B	108.2	C10—C11—N3	118.8 (4)
C1—C6—C5	107.1 (5)	C12—C11—N3	118.6 (3)
C1—C6—H6A	110.3	C13—C12—C11	116.7 (3)
C5—C6—H6A	110.3	C13—C12—H12	121.6
C1—C6—H6B	110.3	C11—C12—H12	121.6
C5—C6—H6B	110.3	C12—C13—C14	123.1 (3)
H6A—C6—H6B	108.5	C12—C13—N4	119.0 (3)
N1—C1'—C6'	110.4 (8)	C14—C13—N4	117.9 (3)
N1—C1'—C2'	115.0 (8)	C13—C14—C9	118.3 (3)
C6'—C1'—C2'	109.4 (6)	C13—C14—H14	120.8
N1—C1'—H1B	107.3	C9—C14—H14	120.8
C6'—C1'—H1B	107.3		
			
C7—N1—C1—C6	147.6 (6)	C8—N2—C7—N1	−0.6 (5)
C1'—N1—C1—C6	134 (2)	C8—N2—C7—S1	−179.3 (3)
C7—N1—C1—C2	−91.4 (9)	C7—N2—C8—O1	0.6 (6)
C1'—N1—C1—C2	−105 (2)	C7—N2—C8—C9	−179.1 (3)
N1—C1—C2—C3	177.3 (7)	O1—C8—C9—C10	−31.7 (5)
C6—C1—C2—C3	−62.8 (10)	N2—C8—C9—C10	148.0 (3)
C1—C2—C3—C4	57.7 (10)	O1—C8—C9—C14	145.0 (4)
C2—C3—C4—C5	−56.9 (9)	N2—C8—C9—C14	−35.3 (4)
C3—C4—C5—C6	57.5 (9)	C14—C9—C10—C11	1.7 (5)
N1—C1—C6—C5	−175.5 (8)	C8—C9—C10—C11	178.6 (3)
C2—C1—C6—C5	63.9 (10)	C9—C10—C11—C12	−0.5 (5)
C4—C5—C6—C1	−59.6 (10)	C9—C10—C11—N3	179.1 (3)
C7—N1—C1'—C6'	141.7 (5)	O3—N3—C11—C10	179.4 (4)
C1—N1—C1'—C6'	−49.4 (18)	O2—N3—C11—C10	1.2 (5)
C7—N1—C1'—C2'	−94.0 (7)	O3—N3—C11—C12	−1.1 (5)
C1—N1—C1'—C2'	74.9 (19)	O2—N3—C11—C12	−179.3 (4)
N1—C1'—C2'—C3'	−66.7 (10)	C10—C11—C12—C13	−0.8 (5)
C6'—C1'—C2'—C3'	58.1 (11)	N3—C11—C12—C13	179.6 (3)
C1'—C2'—C3'—C4'	−59.8 (12)	C11—C12—C13—C14	0.9 (5)
C2'—C3'—C4'—C5'	58.6 (13)	C11—C12—C13—N4	−179.4 (3)
C3'—C4'—C5'—C6'	−57.3 (14)	O5—N4—C13—C12	172.3 (3)
C4'—C5'—C6'—C1'	57.8 (13)	O4—N4—C13—C12	−7.0 (5)
N1—C1'—C6'—C5'	69.9 (10)	O5—N4—C13—C14	−8.0 (5)
C2'—C1'—C6'—C5'	−57.5 (11)	O4—N4—C13—C14	172.6 (3)
C1—N1—C7—N2	177.5 (5)	C12—C13—C14—C9	0.3 (5)
C1'—N1—C7—N2	−177.5 (5)	N4—C13—C14—C9	−179.4 (3)
C1—N1—C7—S1	−3.8 (7)	C10—C9—C14—C13	−1.6 (5)
C1'—N1—C7—S1	1.1 (6)	C8—C9—C14—C13	−178.3 (3)

### Hydrogen-bond geometry (Å, °)

**Table:**

*D*—H···*A*	*D*—H	H···*A*	*D*···*A*	*D*—H···*A*
N1—H1···O1	0.88	1.89	2.639 (4)	142
N1—H1'···O1	0.88	1.99	2.639 (4)	130
N2—H2···S1^i^	0.88	2.65	3.449 (3)	152

Symmetry codes: (i) −*x*+1, −*y*+1, −*z*+1.
